# Supplementary material for: Real-World Goal Setting and Use of Outcome Measures According to the International Classification of Functioning, Disability and Health: A European Survey of Physical Therapy Practice in Multiple Sclerosis
Source: Int J Environ Res Public Health. 2020 Jul 2;17(13):4774. doi: 10.3390/ijerph17134774 (PMC7369767; doi:10.3390/ijerph17134774)
Supplement: Supplementary file 1 [file ijerph-17-04774-s001.pdf]

Supplementary material to the article: Řasová, K.; Martinková, P.; Soler, B.; Freeman, J.; Cattaneo, D.; Jonsdottir, J.; Smedal, T.; Henze, T.; Romberg, A.; Santoyo-Medina, C.; Feys, P. Real-world goal setting and use of outcome measures according to the International Classification of Functioning, Disability and Health: A European survey of physical therapy practice in multiple sclerosis. *International Journal of Environmental Research and Public Health.* 2020.

Table S1. Body structures and functions and activities/participation in an ICF perspective.

**Survey label**	**ICF domain**	**ICF short name**	**ICF Code**
General disability (Assessment only)	Body functions	Body functions	b
Exercise tolerance/ Physical Fitness	Exercise tolerance functions	Exercise tolerance function	b455
Gait/wheelchair pattern functions	Gait pattern function	Gait pattern function	b770
Muscle power function	Muscle power function	Muscle power function	b730
Trunk control	Power of muscles of the trunk / Tone muscles of the trunk	Power/Tone muscles of the trunk	b7305/7355
Ataxia and tremor	Tremor and ataxia	Tremor and ataxia	b750/755/760/765/770
Muscle tone	Muscle tone function	Muscle tone function	b735
Visual function	Seeing functions	Seeing functions	b210
Oculomotor function	Oculomotor function	Oculomotor function	b2152
Mental functions	Mental functions	Mental functions	b1
Psychological functions	Emotional functions	Emotional functions	b152
Fatigue	Fatigability-Energy level	Fatigability-Energy level	b4552/1300
Pain	Pain	Pain	b280-289
Bladder control	Urinary functions	Urinary functions	b610-639
Bowel control	Defecation functions	Defecation functions	b525
Sex functions	Genital and reproductive functions	Genital-Reproductive functions	b640-679
Mobility (walking, wheelchair handling)	Walking and moving	Walking and moving	d450-469
Using arms and hands	Hand and arm use	Hand and arm use	d445
Changing and maintaining body position	Changing and maintaining body position	Changing/Maintaining position	d410/429
Self care	Self-care	Self-care	d5
Domestic life	Domestic life	Domestic life	d6
Leisure and work-related activities (sport, exercises, social act,..)	Recreation and leisure / Work and employment	Recreation-Leisure / Work-Employment	d920/840-859
Quality of life	Major life areas	Major life areas	d8

Table S2. Outcome measures used in assessment of body structures and functions (A.) and activities/participation (B.) in MS patients

**A. Body structures and functions**							
**Abbreviation**	**Outcome measure**	**Total**	**East**	**North**	**South**	**West**	**p value**
**Body functions (ICF classification: b)**							
EDSS	Expanded Disability Status Scale	81 (40.9%)	12 (40%)	18 (30%)	36 (41.4%)	15 (71.4%)	0.240
MSFC	Multiple Sclerosis Functional Composite	35 (17.7%)	6 (20%)	6 (10%)	14 (16.1%)	9 (42.9%)	0.460
GNDS	The Guy's Neurological Disability Scale	16 (8.1%)	4 (13.3%)	5 (8.3%)	6 (6.9%)	1 (4.8%)	0.931
SNRS	Scripps Neurological Rating Scale	4 (2%)	1 (3.3%)	0 (0%)	3 (3.4%)	0 (0%)	0.619
**Mental/Emotional functions (ICF classification: b1/152)**							
MMSE	Mini-Mental State Examination	51 (29.3%)	9 (34.6%)	10 (18.5%)	26 (33.3%)	6 (37.5%)	0.519
BDIS	Behavior Disorders Identification Scale	20 (11.5%)	3 (11.5%)	4 (7.4%)	9 (11.5%)	4 (25%)	0.574
PASAT	Hospital Anxiety and Depression Scale	18 (10.3%)	4 (15.4%)	5 (9.3%)	5 (6.4%)	4 (25%)	0.490
HADS	Paced Auditory Serial Addition Test	18 (10.3%)	1 (3.8%)	7 (13%)	8 (10.3%)	2 (12.5%)	0.387
MHI	Symbol Digit Modalities Test	14 (8%)	2 (7.7%)	4 (7.4%)	6 (7.7%)	2 (12.5%)	0.792
SDMT	Mental Health Inventory	11 (6.3%)	1 (3.8%)	4 (7.4%)	5 (6.4%)	1 (6.2%)	0.460
**Seeing and Oculomotor Functions (ICF classification: b210/2152)**							
L-CLA	Low Contrast Letter Acuity	9 (5%)	3 (9.1%)	2 (3.8%)	4 (5.4%)	0 (0%)	0.533
Snellen / Sloan	Snellen chart / Sloan chart	6 (3.4%)	3 (9.1%)	1 (1.9%)	2 (2.7%)	0 (0%)	0.616
Oculomotor ICARS	Oculomotor International Cooperative Ataxia Rating Scale	6 (3.4%)	3 (9.1%)	0 (0%)	3 (4.1%)	0 (0%)	0.387
OMS	Ocular Motor Score	5 (2.8%)	2 (6.1%)	0 (0%)	3 (4.1%)	0 (0%)	0.387
**Vestibular functions (ICF classification: b235)**							
BBS	Berg Balance Scale	124 (60.5%)	7 (21.2%)	56 (88.9%)	46 (52.3%)	15 (71.4%)	**0.004**
Number of falls	Number of falls	85 (41.5%)	12 (36.4%)	36 (57.1%)	31 (35.2%)	6 (28.6%)	**0.040**
TMT	Tinetti Mobility Test	57 (27.8%)	2 (6.1%)	8 (12.7%)	37 (42%)	10 (47.6%)	**0.004**
DGI	Dynamic Gait Index	39 (19%)	4 (12.1%)	20 (31.7%)	12 (13.6%)	3 (14.3%)	**0.006**
Stabilometry	Stabilometry	38 (18.5%)	11 (33.3%)	2 (3.2%)	18 (20.5%)	7 (33.3%)	**0.004**
ABC	Activities-specific Balance Confidence Scale	20 (9.8%)	1 (3%)	11 (17.5%)	7 (8%)	1 (4.8%)	0.205
DHI	Dizziness Handicap Inventory	10 (4.9%)	0 (0%)	5 (7.9%)	5 (5.7%)	0 (0%)	0.387
**Fatigability/energy level/Pain (ICF classification: b280-289/1300/4552)**							
Pain rating scales	Modified Fatigue Impact Scale	97 (46.9%)	13 (38.2%)	34 (53.1%)	42 (47.7%)	8 (38.1%)	0.164

MFIS	Modified Fatigue Impact Scale	60 (29%)	4 (11.8%)	15 (23.4%)	32 (36.4%)	9 (42.9%)	0.129
FSS	Fatigue Severity Scale	54 (26.1%)	3 (8.8%)	23 (35.9%)	21 (23.9%)	7 (33.3%)	**0.011**
FSMC	Fatigue Scale for Motor and Cognitive functions	19 (9.2%)	4 (11.8%)	3 (4.7%)	10 (11.4%)	2 (9.5%)	0.937
**Exercise tolerance function (ICF classification: b455)**							
Heart rate	Heart rate	110 (54.5%)	15 (45.5%)	36 (57.1%)	44 (51.8%)	15 (71.4%)	**0.161**
RPE	Borg Rating of Perceived Exertion	101 (50%)	8 (24.2%)	47 (74.6%)	35 (41.2%)	11 (52.4%)	**0.004**
Oxygen consumption	Oxygen consumption	31 (15.3%)	4 (12.1%)	8 (12.7%)	17 (20%)	2 (9.5%)	0.164
Spirometry	Spirometry	25 (12.4%)	6 (18.2%)	6 (9.5%)	9 (10.6%)	4 (19%)	0.733
**Urinary/Defecation/Genital-reproductive function (ICF classification: b610-639/525/640-679)**							
Bladder & bowel control	Bladder & bowel control	12 (7.6%)	2 (7.4%)	0 (0%)	8 (11.3%)	2 (11.8%)	0.138
Bladder & bowel function	Bladder & bowel function	12 (7.6%)	2 (7.4%)	0 (0%)	8 (11.3%)	2 (11.8%)	0.164
SSS	Sexual Satisfaction Scale	7 (4.4%)	3 (11.1%)	0 (0%)	3 (4.2%)	1 (5.9%)	0.387
**Muscle power function (ICF classification: b730)**							
MFT	Manual Function Test	85 (41.1%)	15 (44.1%)	37 (57.8%)	25 (28.4%)	8 (38.1%)	0.164
Endurance	Endurance	75 (36.2%)	6 (17.6%)	30 (46.9%)	33 (37.5%)	6 (28.6%)	0.129
Dynamometry	Dynamometry	45 (21.7%)	8 (23.5%)	18 (28.1%)	13 (14.8%)	6 (28.6%)	0.552
MRC	Medical Research Council	42 (20.3%)	4 (11.8%)	11 (17.2%)	16 (18.2%)	11 (52.4%)	**0.014**
Motricity index	Motricity index	42 (20.3%)	3 (8.8%)	5 (7.8%)	23 (26.1%)	11 (52.4%)	**0.004**
Motor club assessment	Motor club assessment	13 (6.3%)	3 (8.8%)	3 (4.7%)	5 (5.7%)	2 (9.5%)	0.839
**Power/Tone muscles of the trunk (ICF classification: b7305/7355)**							
Trunk control test	Trunk control test	89 (43.4%)	12 (35.3%)	21 (32.8%)	42 (48.3%)	14 (70%)	**0.017**
Trunk impairment scale	Trunk impairment scale	51 (24.9%)	6 (17.6%)	17 (26.6%)	17 (19.5%)	11 (55%)	0.205
**Muscle tone function (ICF classification: b735)**							
MAS	Modified Ashworth Scale	145 (70.7%)	20 (60.6%)	51 (82.3%)	60 (67.4%)	14 (66.7%)	0.164
MT rating scales	Rating scale, e.g. numerical rating scale, visual analogue scale	91 (44.4%)	11 (33.3%)	32 (51.6%)	38 (42.7%)	10 (47.6%)	0.577
Joint angle kinematic	Joint angle kinematic	35 (17.1%)	2 (6.1%)	12 (19.4%)	18 (20.2%)	3 (14.3%)	0.460
Tardieu scale	Tardieu scale	25 (12.2%)	4 (12.1%)	7 (11.3%)	9 (10.1%)	5 (23.8%)	0.338
SFS	Spasm Frequency Scale	25 (12.2%)	3 (9.1%)	8 (12.9%)	10 (11.2%)	4 (19%)	>0.999
Electromyography	Electromyography	19 (9.3%)	4 (12.1%)	2 (3.2%)	11 (12.4%)	2 (9.5%)	**0.028**
MSSS-88	88-item Multiple Sclerosis Spasticity Scale	14 (6.8%)	0 (0%)	4 (6.5%)	7 (7.9%)	3 (14.3%)	0.161

**Tremor and Ataxia (ICF classification: b7651/750/755/760/765/770)**							
SARA	Scale for Assessment and Rating of Ataxia	13 (6.4%)	2 (5.9%)	3 (4.8%)	8 (9.3%)	0 (0%)	0.327
ICARS	International Cooperative Ataxia Rating Scale	12 (5.9%)	4 (11.8%)	2 (3.2%)	5 (5.8%)	1 (4.8%)	0.164
FTM	Fahn-Tolosa-Marín scale	11 (5.4%)	3 (8.8%)	2 (3.2%)	5 (5.8%)	1 (4.8%)	0.460
**Gait pattern function (ICF classification: b770)**							
Spatio-temporal params	Spatio-Temporal parameters	129 (61.7%)	17 (50%)	47 (73.4%)	50 (55.6%)	15 (71.4%)	0.460

**B. Activities and participation**							
**Abbrevation**	**Outcome measure**	**Total**	**East**	**North**	**South**	**West**	**p value**
**Hand and arm use (ICF classification: d445)**							
9HPT	Nine Hole Peg Test	56 (27.5%)	7 (21.2%)	22 (35.5%)	20 (22.7%)	7 (33.3%)	**0.047**
BBT	Box and Block Test	17 (8.3%)	2 (6.1%)	4 (6.5%)	6 (6.8%)	5 (23.8%)	0.339
DASH	Disabilities of the Arm, Shoulder and Hand questionnaire	17 (8.3%)	2 (6.1%)	1 (1.6%)	10 (11.4%)	4 (19%)	0.151
ARAT	Action Research Arm Test	16 (7.8%)	1 (3%)	2 (3.2%)	6 (6.8%)	7 (33.3%)	**0.013**
PPT	Purdue Pegboard Test	13 (6.4%)	3 (9.1%)	0 (0%)	7 (8%)	3 (14.3%)	0.490
CAHAI-7	Chedoke Arm and Hand Activity Inventory	13 (6.4%)	1 (3%)	1 (1.6%)	10 (11.4%)	1 (4.8%)	0.073
WMFT	Wolf Motor Function Test	7 (3.4%)	0 (0%)	0 (0%)	6 (6.8%)	1 (4.8%)	0.291
TEMPA	Test Evaluant la Performance des Membres supérieurs des Personnes âgées	6 (2.9%)	0 (0%)	0 (0%)	5 (5.7%)	1 (4.8%)	0.099
**Walking - Moving (ICF classification: d450-469)**							
TUG	Timed Up and Go Test	116 (55.8%)	13 (39.4%)	50 (78.1%)	38 (42.2%)	15 (71.4%)	**0.004**
6MWT	6-Minute Walk test	109 (52.4%)	5 (15.2%)	55 (85.9%)	32 (35.6%)	17 (81%)	**0.004**
10MWT normal	10- Meter Walk test normal speed	93 (44.7%)	9 (27.3%)	41 (64.1%)	33 (36.7%)	10 (47.6%)	**0.015**
10MWT maximal	10-Meter Walk test maximal speed	87 (41.8%)	8 (24.2%)	38 (59.4%)	32 (35.6%)	9 (42.9%)	**0.014**
T25FW	Timed 25-Foot Walk	74 (35.6%)	9 (27.3%)	26 (40.6%)	27 (30%)	12 (57.1%)	0.161
2MWT	2-Minute Walk Test	69 (33.2%)	3 (9.1%)	26 (40.6%)	27 (30%)	13 (61.9%)	**0.006**
MSWS-12	12 Item Multiple Sclerosis Walking Scale	48 (23.1%)	1 (3%)	28 (43.8%)	15 (16.7%)	4 (19%)	**0.004**
DGI	Dynamic Gait Index	38 (18.3%)	4 (12.1%)	16 (25%)	15 (16.7%)	3 (14.3%)	0.148
RMI	Rivermead Mobility Index	20 (9.6%)	0 (0%)	5 (7.8%)	13 (14.4%)	2 (9.5%)	0.205
FAC	Functional Ambulation Category	20 (9.6%)	2 (6.1%)	4 (6.2%)	7 (7.8%)	7 (33.3%)	**0.006**

RMA	Rivermead Mobility Index	15 (7.2%)	0 (0%)	6 (9.4%)	8 (8.9%)	1 (4.8%)	0.164
FSQ	Functional Status Questionnaire	11 (5.3%)	0 (0%)	4 (6.2%)	7 (7.8%)	0 (0%)	0.151
AI	Hauser Ambulation Index	9 (4.3%)	0 (0%)	0 (0%)	8 (8.9%)	1 (4.8%)	**0.034**
**Self-care (ICF classification: d5)**							
BI	Barthel Index	85 (43.4%)	9 (28.1%)	9 (14.8%)	60 (72.3%)	7 (35%)	**0.004**
FIM	Functional Independence Measure	65 (33.3%)	6 (18.8%)	9 (14.8%)	38 (46.3%)	12 (60%)	**0.004**
ISS	Injury Severity Score	16 (8.2%)	0 (0%)	0 (0%)	13 (15.9%)	3 (15%)	**0.006**
MSES	Moorong Self-Efficacy Scale	14 (7.2%)	1 (3.1%)	3 (4.9%)	9 (11%)	1 (5%)	0.460
FAMS	Functional Assessment of Multiple Sclerosis	13 (6.7%)	0 (0%)	8 (13.1%)	5 (6.1%)	0 (0%)	**0.047**
FSQ	Functional Status Questionnaire	9 (4.6%)	1 (3.1%)	1 (1.6%)	7 (8.5%)	0 (0%)	0.164
**Major life areas (ICF classification: d8)**							
MSIS-29	Multiple Sclerosis Impact Scale-29	30 (15.2%)	1 (3.2%)	13 (22%)	13 (14.8%)	3 (15%)	**0.011**
SF-12 / SF-36	12 Item Short Form Survey/ 36 Item Short Form Survey	22 (11.1%)	1 (3.2%)	7 (11.9%)	12 (13.6%)	2 (10%)	0.387
MS QoL	Multiple Sclerosis Quality of Life	21 (10.6%)	1 (3.2%)	4 (6.8%)	14 (15.9%)	2 (10%)	0.464
LHS	London Handicap Scale	8 (4%)	0 (0%)	0 (0%)	8 (9.1%)	0 (0%)	0.338
SIP-68	Sickness Impact Profile 68	7 (3.5%)	0 (0%)	0 (0%)	6 (6.8%)	1 (5%)	0.346
NHP	Nottingham Health Profile	5 (2.5%)	0 (0%)	0 (0%)	4 (4.5%)	1 (5%)	0.387
**Recreation-Leisure/Work-Employment (ICF classification: d920/d840-59)**							
FSE	Functional Status Examination	13 (6.7%)	2 (6.7%)	1 (1.7%)	9 (10.6%)	1 (5%)	0.327
FAI	Frenchay Activities Index	6 (3.1%)	0 (0%)	2 (3.4%)	4 (4.7%)	0 (0%)	0.240
MSSS	Multiple Sclerosis Severity Score	6 (3.1%)	0 (0%)	0 (0%)	6 (7.1%)	0 (0%)	0.460
ESS	Epworth Sleepiness Scale	4 (2.1%)	0 (0%)	0 (0%)	4 (4.7%)	0 (0%)	0.481

Note: Numbers of respondents (physiotherapists) who use given outcome measure at least “sometimes” in assessing their MS patients. Percentages are calculated from number of respondents from given region who assess given body function / activity. The p values were adjusted for multiple comparisons, significant p values are highlighted in bold.
